# Cancer stem cells, plasticity, and drug resistance

**DOI:** 10.20517/cdr.2019.112

**Published:** 2020-02-21

**Authors:** Maria Chiara Lionetti, Maria Rita Fumagalli, Caterina A. M. La Porta

**Affiliations:** ^1^Center for Complexity and Biosystems, Department of Environmental Science and Policy, University of Milan, via Celoria 26, Milano 20133, Italy.; ^2^CNR - Consiglio Nazionale delle Ricerche, Istituto di Biofisica, Via de Marini 6, Genova 16149, Italy.; ^#^Authors contributed equally.

**Keywords:** Melanoma, drug resistance, epithelial-mesenchymal transition, precision medicine, tumor plasticity

## Abstract

Melanoma is a highly aggressive tumor and almost always fatal when metastatic. Herein, we discuss recent findings on the mechanisms of resistance of human cutaneous melanoma. To achieve a precision medicine approach, the heterogeneity and plasticity of tumor cells are two crucial aspects to be investigated in depth. In fact, to understand the mechanisms that cells use to acquire a resistant phenotype after chemotherapy or how resistant cells inside the tumor are selected, it is the most important issue for a successful therapy. Since new therapeutic strategies are trying to go in this direction, we discuss here the state of the art of the research and the clinical impact of these strategies. We also discuss and suggest future research directions to develop approaches able to define the best concentration and time of exposure of the drug or the cocktails of drugs for each specific patient based on his/her biological features.

## Current therapeutic strategies for the treatment of human cutaneous melanoma

**Figure 1:**
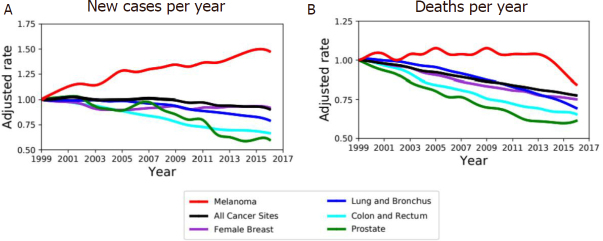
Melanoma incidence and death rate. A: age-adjusted rate of new cancer cases diagnosed in USA between 1999 and 2016 normalized over corresponding rate in 1999 for both sexes. Incidence is increasing in melanoma (red) compared to other type of tumors (colors as in legend). B: Age-adjusted death rate, for both sexes, between 1999 and 2016 in USA normalized over corresponding rate in 1999 for melanoma and top-rated cancers by rates of cancer deaths

Melanoma arises from mutated melanocytes, the pigment producing cells. Although melanoma is a rare tumor, occurring in about 1% of all skin malignant tumors, it represents almost 2% of all cancer deaths worldwide^[1]^; the survival rate is strictly related to the stage of the tumor and thus to the ability to perform an early diagnosis^[2,3]^ [Figure 1]. Furthermore, the age-adjusted rate of new cases reported in the USA between 1999 and 2016 shows an important increase of new cases of melanoma per year with respect to others kinds of cancer such as lung, breast, and colon cancers [Figure 1]. The overall survival is higher in the case of localized disease, but patients with metastatic melanoma show a very poor prognosis, with a median survival rate ranging 3-6 months^[2-4]^. While low-grade primary tumors are usually successfully treated by surgical excision, systemic treatment of advanced metastatic disease treated with chemotherapy shows a low response rate and generally no overall survival rate improvement^[5]^.

Cutaneous melanoma is characterized by a series of peculiar somatic genetic alterations, many of which are common in others tumors such as genes responsible for the control of cell cycle and proliferation, metabolism, growth, and apoptosis that typically lead to the deregulation of mitogen-activated protein kinase (MAPK) and the phosphoinositol-3-kinase (PI3K)/AKT pathways^[6,7]^. The most frequently mutated gene is *BRAF*, and in particular the missense mutation V600E is typical of this kind of tumor since it is the most frequent mutation occurring in melanoma^[8-10]^ while NRAS activating mutations have been detected in a small percentage of these tumor cases^[8,11]^. BRAF is a serine-threonine kinase involved in the RAF/MEK/MAPK pathway controlling through ERK1/2 cellular proliferation, survival, and differentiation^[9]^. Notably, NRAS and BRAF mutations are generally mutually exclusive; only in a minor proportion of patients the coexistence of both genetic alterations is reported^[9,12]^. According to Genomic Data Commons portal data, mutations on BRAF and NRAS genes taken together affect about 10% of all the considered cancers but are detected in about 52% and 27%, respectively, of the melanoma patients in this cohort. Additionally, genetic alterations of telomerase reverse transcriptase promoter (TERT) and cyclin dependent kinase inhibitor 2A (CDK2A) or phosphatase and tensin homolog (PTEN) loss-of-function have been frequently observed in advanced melanoma^[13-18]^.

Current therapeutic approaches for cutaneous melanoma include surgical resection, chemotherapy, photodynamic therapy, immunotherapy, biochemotherapy, and targeted therapy, depending on the features of the tumor such as its localization, stage, and genetic profile. Chemotherapy combinations have been shown to improve the clinical response, but the overall survival does not change significantly^[20]^. Dacarbazine, approved in 1974 by Federal and Drug Administration (FDA), is the standard drug used for metastatic melanoma. Temozolomide, which is an oral pro-drug of the active metabolite of dacarbazine, is used in advanced melanoma and it seems to improve the median progression-free survival but not the overall survival^[21,22]^. Electrochemotherapy is a technique that combines the use of cytotoxic drugs such as bleomycin and cisplatin with high-intensity electric pulses, which facilitate the drug delivery inside the cells^[23,24]^. Light-based therapy is a promising adjuvant therapy useful for palliative treatment in advanced metastatic melanomas^[25]^.

**Figure 2:**
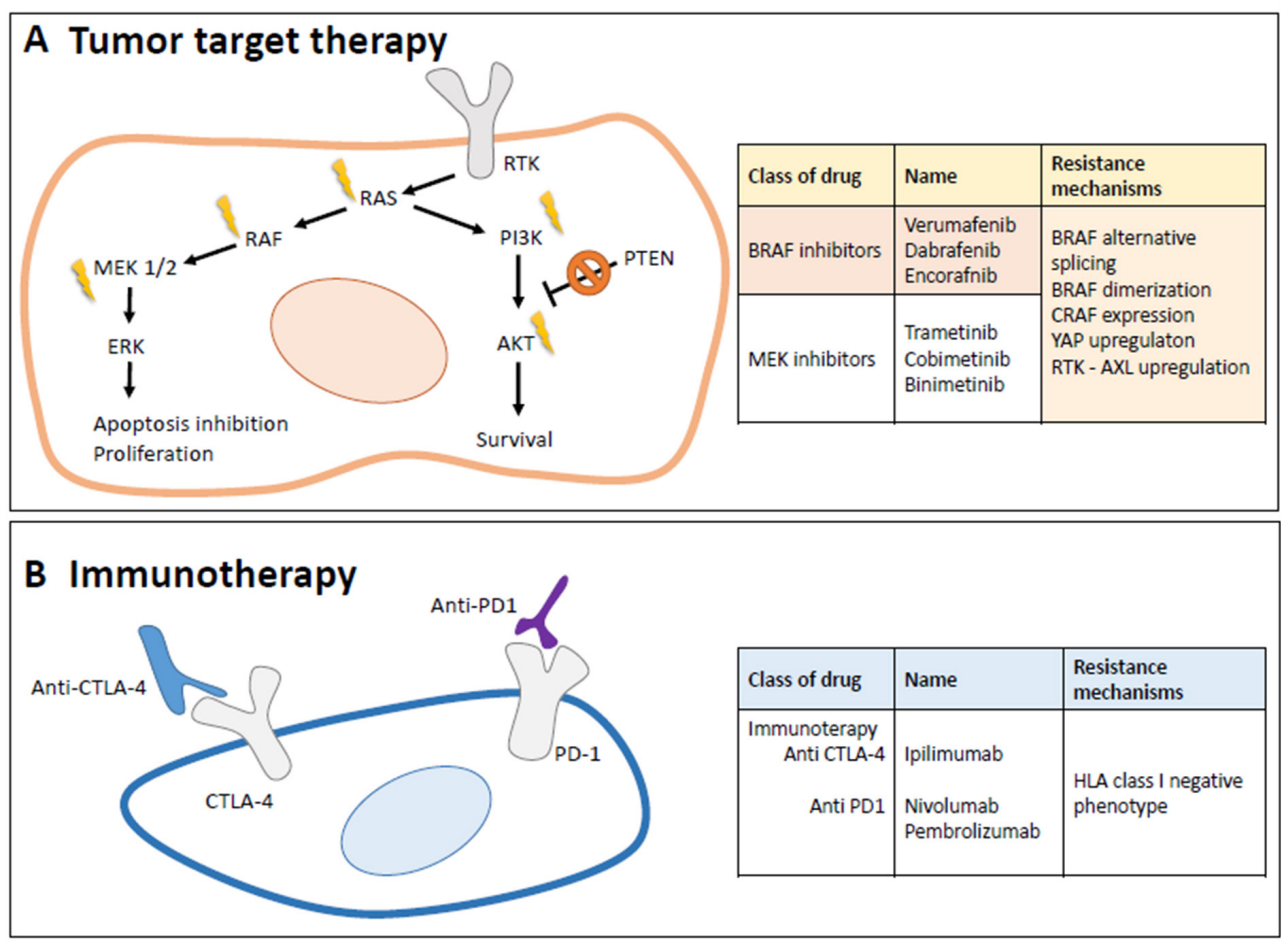
A: tumor target therapy. Simplified schematic of the key molecular component of MAPK and PI3K/Akt signaling pathway related to melanoma tumorigenesis and targeted inhibitors of representative drugs and therapies with the main resistant mechanisms. B: immunotherapy. The target of the tumor cell environment, the most important drugs, and the main resistance mechanisms. HLA: human leukocyte antigen

Immunotherapy is mainly based on the frequent presence of chronic inflammation and of immune cells inside the tumor^[26]^. The possibility to target the immunogenic tumor microenvironment is nowadays one of the more promising strategies for a successful cancer treatment. Regarding cutaneous melanoma, there are immunotherapies approved by FDA (e.g., nivolumab, pembrolizumab, and gp100 vaccine). Nivolumab and pembrolizumab, approved for the treatment of metastatic melanoma, re anti-Programmed cell Death protein (PD1) antibodies that block the interaction between PD-1, which is a membrane antigen, and its ligand programmed death-ligand 1 (PD-L1)/PD-L2. The blockade of the interaction between this ligand and its receptor induces antitumor activity, showing a reduction of tumor progression through the modulation of the immune system^[27]^. Another interesting drug is ipilimumab, which is an anti-cytotoxic T-lymphocyte-associated protein 4 antibody that acts as a receptor antagonist, enhancing pro-inflammatory T-cell cytokine production and promoting clonal T-cell expansion^[28,29]^. In Figure 2, we report a scheme of the pathways on which these drugs work.

Gp100 is a glycoprotein expressed by melanoma cells with few exceptions (healthy epidermal melanocytes and retina) and it is recognized by cytotoxic T cells (CTL). The administration of gp100 epitopes enhances CTLs activity; however, it is reported to have limited clinical benefits and it is used as adjuvant therapy only^[30]^.

Biochemotherapy is a combination of chemotherapy and immunotherapy. In fact, some conventional chemotherapies may act partially through immune-stimulatory mechanisms^[31]^. The most common use of biochemotherapy is the combination of dacarbazin, cisplatin, and vinblastine with interleukine-2 (IL-2) and interferon (IFN)α-2b as immunoregulators.

Most cutaneous melanomas are treated with targeted therapy, since about 70% of these tumors express specific mutations related to key signaling pathways (e.g., BRAF V600E) [Figure 2]. Targeted therapy by using small molecule inhibitors or antibodies affecting those mutated proteins which play a critical role in the progression of the tumor [Figure 2] are discussed in the next section.

## Genetic and epigenetic mechanisms of resistance

Melanoma is a highly resistant tumor. The appearance of resistance after chemotherapy or the presence of intrinsic resistance leads to great difficulties in devising an effective and durable therapy and, finally, to a poor survival of the patients, in particular when they are already metastatic. In recent years, many studies were designed with the aim to understand the molecular basis of resistance. We herein discuss the main biological mechanisms displayed by melanoma to become resistant to current therapies.

Treatment of advanced BRAF V600E mutant melanoma using a BRAF inhibitor or its combination with a MEK inhibitor typically elicits only partial response. It has been reported that new genetic alterations arise in patients carrying BRAF mutation when treated with anti-BRAF antibody as well as in patients displaying both BRAF and MEK mutations and treated with inhibitors for both factors^[32,33]^. In particular, it has been reported that one of the main mechanisms of resistance is the reactivation of MAPK signaling^[33]^. Moreover, in a recent paper, a comparison between the transcriptomes of melanoma patient-derived tumors regressing after MAPK inhibitor (MAPKi) treatment with respect to MAPKi-induced temporal transcriptomic states showed that residual melanoma on MAPKi therapy displays adaptive transcriptomic, epigenomic, and immune-regulomic alterations^[34]^.

Hannan and coworkers observed that an incorrect analysis of the melanoma polyclonal population affects the choice of the therapy^[35]^. This aspect is relevant for melanoma since drug therapy is usually applied when the disease is in advanced state^[2]^. The deletion or loss of function of PTEN is also quite common in drug-resistant melanoma, reactivating PI3K/ATK pathway in a MAPK-independent manner^[36-38]^. On the other hand, transient resistance can be induced by compensatory changes in gene expression such as the upregulation of the receptor of tyrosine kinases, the overexpression of CRAF, or the amplification or truncation of BRAF gene^[37,39-41]^.

The high heterogeneity of the tumor cells and their plasticity lead to the possibility that the same drug might induce the switch to slow-cycling resistant phenotype associated to high melanocyte inducing transcription factor levels and to a mesenchymal-like phenotype^[42-46]^. Early adaptation involving transcriptome reprogramming seems to be particularly relevant even at long time scale, allowing the tumor to survive until a genetic mutation and permanent resistance mechanism is acquired^[43,47]^. Interestingly, melanoma cells can display profound transcriptional variability at the level of single cell that can involve the transcription of a number of resistance markers at high level in a very small percentage of cells^[48]^. The presence of a drug can, therefore, induce an epigenetic reprogramming in these cells, converting the transient transcriptional state into a stable one^[48]^.

Other important actors of drug resistance in melanoma are non-coding RNAs^[49-51]^. In this context, the use of combined and coadjuvant therapies has been proposed to avoid successive treatment failures due to the acquisition of a cross-resistance or changes in tumor environment^[52-57]^. Moreover, the tumor niche can play an important role and a long-term success of targeted therapies seems to be strictly related to a favorable microenvironment and immunologic signature^[54,58,59]^. In this connection, a recent paper shows that the development of drug resistance to anti-BRAF treatment is dominated by a dynamic deregulation of a large population of microRNA (miRNA)^[60]^. The latter leads to the alteration of the intrinsic proliferation and survival pathways, enhancing proinflammatory and proangiogenic cues^[60]^.

## Role of immunity in resistance

Chronic inflammation is a hallmark of cancer^[61-63]^. Innate and adaptive immune responses contribute to select aggressive clones, stimulating cancer cell proliferation and migration^[64]^. Natural killers and CTL can recognize and eliminate the immunogenic cancer cells and in this way less immunogenic cells are selected^[65]^. Tumor associated macrophages and neutrophils can also promote angiogenesis and lymphangiogenesis as well as cancer cell proliferation and epithelial-mesenchymal transition (EMT) by secreting a set of stimulating cytokines^[66-68]^. However, the same tumor cells can secrete immunosuppressive factors, controlling the immune response^[68-70]^. Tumor-associated endothelial cells also contribute to make cancer physically inaccessible to the immune system by increasing deposition of factors that, on the one hand, confer a higher stiffness of the extracellular matrix and, on the other hand, prevent immune infiltration in the tumor tissue and favor tumor cell proliferation^[69,71]^. In light of these findings, several different immunotherapeutic strategies have been developed. Cytokines with immunomodulatory, antiangiogenic, anti-proliferative, and antitumor activities, such as IFNs and IL-2, have been combined with chemotherapy but with less satisfying results^[72]^. Immune-checkpoint inhibitors, a class of target-specific drugs that interfere with critical inhibitory signaling pathways promoting immune-mediated target of tumor cells (e.g., ipilimumab and nivolumab), gave more successful results^[73]^.

Adoptive T-cells transfer therapy is, at the moment, one of the personalized and effective treatment methods available for the management of metastatic melanoma. In this case, tumor-infiltrating lymphocytes directly derived from the patients or genetically engineered melanoma-specific T-cells are expanded *ex-vivo* and then injected into the patient^[74]^. Although the complex anti-tumor mechanism triggered by this therapeutic approach has not yet been fully elucidated, the obtained results are very promising. Adoptive T-cells transfer therapy has been reported to be associated with complete and durable responses also in metastatic melanomas^[75]^. This approach results effective not only alone but also in combination with other standard therapies for melanoma management^[76]^.

## Phenotypic plasticity and drug resistance

Cancer is highly heterogeneous. This fact brings many important consequences: there is profound variation between different individuals with the same type of cancer as well as a high grade of genetic and phenotypical variability in the cancer cell population of a specific subject. The different phenotypes of tumor cells are due not only to genetic and epigenetic intratumor heterogeneity^[77]^ but also to epigenetic changes due to the impact of the environment. It has been reported that genetically homogeneous tumor cells show a remarkable diversity in their response to therapy or other environmental stimuli^[78,79]^. Epigenetic gene regulation at the molecular level from DNA methylation, post-translational modification of histones, non-coding RNAs, and chromatin remodeling are the most common mechanisms contributing to cellular epigenetic heterogeneity. For a cancer, the robustness of the system comes from the ability of the cells to adapt to another environment, having the capacity to evolve into new cellular ecosystems. The ability of cancer cells to adapt to critical changes in the environment leads to the difficulty in finding a successful strategy. The main genetic mechanism that contributes to the ability of cancer to adapt to different microenvironments is genetic destabilization^[80,81]^. Furthermore, differences in tumor cell metabolism, due to genetic mutations and/or altered microenvironment, have a direct impact on epigenetic changes^[82]^. In this connection, our group recently demonstrated that human melanoma cells can change their phenotype, expressing EMT markers dynamically, thanks to a complex network of miRNAs^[83]^. The direct and more important consequence of these findings is that the cells show an intrinsic ability to dynamically change the phenotype, depending on the environment^[83]^. Similar results were published recently for breast cancer^[84]^.

The impact of plasticity of tumor on drug resistance is a key factor and is crucial to develop new therapeutic strategies. In this connection, a recent interesting paper describes the dynamics of single melanoma cells after the treatment with a drug and shows that the cells reprogram to a stable resistant state^[48]^. The reprogramming involves the loss of SOX-10, which mediates differentiation, and the activation of Jun-Activator protein 1 and TEAD^[48]^.

## Conclusion and perspectives

Plasticity of tumor cells including melanoma is a critical issue for a successful therapeutic strategy. The ability of tumor cells to change their status using epigenetic mechanisms in dependence of the environment, such as the tumor niche, has been shown to play a critical role in the acquisition of a resistant phenotype in response to a specific drug. In light of these findings, in our opinion, the following will be crucial for precision medicine treatments: (1) knowing the epigenetic profile of each specific tumor of each specific patient before the treatment to start the best therapy; and (2) avoiding the ability of tumor cells to change their phenotype during the treatment, thus acquiring a resistant phenotype, by acting both at the level of the tumor cells and at the level of the tumor niche.

## Declarations

### Copyright

© The Author(s) 2020.
